# The Neurotransmitters Involved in *Drosophila* Alcohol-Induced Behaviors

**DOI:** 10.3389/fnbeh.2020.607700

**Published:** 2020-12-15

**Authors:** Maggie M. Chvilicek, Iris Titos, Adrian Rothenfluh

**Affiliations:** ^1^Department of Psychiatry, University of Utah, Salt Lake City, UT, United States; ^2^Molecular Medicine Program, University of Utah, Salt Lake City, UT, United States; ^3^Neuroscience Graduate Program, University of Utah, Salt Lake City, UT, United States; ^4^Department of Neurobiology and Anatomy, University of Utah, Salt Lake City, UT, United States; ^5^Department of Human Genetics, University of Utah, Salt Lake City, UT, United States

**Keywords:** *Drosophila*, alcohol behavior, neurotransmitter, alcohol abuse, AUD, genetics

## Abstract

Alcohol is a widely used and abused substance with numerous negative consequences for human health and safety. Historically, alcohol's widespread, non-specific neurobiological effects have made it a challenge to study in humans. Therefore, model organisms are a critical tool for unraveling the mechanisms of alcohol action and subsequent effects on behavior. *Drosophila melanogaster* is genetically tractable and displays a vast behavioral repertoire, making it a particularly good candidate for examining the neurobiology of alcohol responses. In addition to being experimentally amenable, *Drosophila* have high face and mechanistic validity: their alcohol-related behaviors are remarkably consistent with humans and other mammalian species, and they share numerous conserved neurotransmitters and signaling pathways. Flies have a long history in alcohol research, which has been enhanced in recent years by the development of tools that allow for manipulating individual *Drosophila* neurotransmitters. Through advancements such as the GAL4/UAS system and CRISPR/Cas9 mutagenesis, investigation of specific neurotransmitters in small subsets of neurons has become ever more achievable. In this review, we describe recent progress in understanding the contribution of seven neurotransmitters to fly behavior, focusing on their roles in alcohol response: dopamine, octopamine, tyramine, serotonin, glutamate, GABA, and acetylcholine. We chose these small-molecule neurotransmitters due to their conservation in mammals and their importance for behavior. While neurotransmitters like dopamine and octopamine have received significant research emphasis regarding their contributions to behavior, others, like glutamate, GABA, and acetylcholine, remain relatively unexplored. Here, we summarize recent genetic and behavioral findings concerning these seven neurotransmitters and their roles in the behavioral response to alcohol, highlighting the fitness of the fly as a model for human alcohol use.

## Introduction

Alcohol is one of the most commonly used and abused psychoactive substances. Approximately 86% of American adults have reported drinking alcohol at some point in their lifetimes (Substance Abuse and Mental Health Services Administration, 2019), and, as of 2018, alcohol use disorder (AUD) affected over 14 million adults in the United States (Substance Abuse and Mental Health Services Administration, 2019). AUD is characterized by an impaired ability to control alcohol use despite negative consequences for personal and public health and safety (Substance Abuse and Mental Health Services Administration, 2018). AUD is also frequently correlated with psychological conditions like anxiety (Grant et al., 2004), depression (Hasin et al., 2005), post-traumatic stress disorder (Marshall et al., 2012), and medical history of an anxiety or mood disorder (Martins and Gorelick, 2011). Alcohol-related behaviors are multifaceted, impacted by numerous environmental and individual factors. Due to these complexities, alcohol may cause problematic use and addiction in some people but have minimal consequences in others.

Research established a genetic basis for alcohol use as early as the 1950s (Amark, 1951). Several genes are associated with problematic alcohol use, and twin studies suggest that AUD is ~50% heritable (Verhulst et al., 2015). Although it is clear that disorders like AUD, which present with behavioral alterations, are influenced by genetics, translating knowledge about genes, cells, and anatomy into a mechanistic understanding of behavior remains one of the biggest challenges in neurobiology. Therefore, the discovery that a genetically tractable organism like *Drosophila melanogaster* (henceforth called *Drosophila* or flies) shows a broad behavioral repertoire facilitated a new chapter of neuroscience research. Flies, like humans and other mammals, modulate their behaviors according to circadian rhythms (Dubowy and Sehgal, 2017), can learn and remember (Cognigni et al., 2018), and show behavioral hallmarks of addiction (Devineni and Heberlein, 2009; Kaun et al., 2012), among other behaviors.

The neurobiological action of alcohol is especially challenging to understand since alcohol does not have a specific target pathway and instead affects pathways intended for other physiological functions (Fadda and Rossetti, 1998). Research is still unraveling how alcohol alters various brain circuits and why some are more susceptible to alcohol than others. Behavior is a useful tool for examining where and how alcohol may be affecting the brain since there are known behavioral outcomes associated with specific circuits and neurotransmitter systems. Given its high rates of use and abuse, understanding the neural and behavioral outcomes of alcohol is critical. Here we will focus on the role of *Drosophila's* neurotransmitter pathways in behavior and how that behavior is affected under the influence of alcohol.

## *Drosophila* as a Model Organism

For over a hundred years, *Drosophila melanogaster*, commonly known as the fruit or vinegar fly, has been a critical model organism for the field of neuroscience (Bellen et al., 2010). Flies have many characteristics that make them an appealing organism in the laboratory: short generation time, low cost, ease of maintenance, and relatively simple genetic and anatomical makeup. *Drosophila* were one of the first organisms for which the genome was fully sequenced (2000), and flies have many genetic similarities to humans, sharing an estimated 62% homology in disease-causing genes (Fortini et al., 2000). The *Drosophila* nervous system consists of ~300,000 neurons making up the brain and thoracic ganglion, which is the fly equivalent of the spinal cord (Freeman, 2015). The majority of small-molecule neurotransmitters responsible for central nervous system (CNS) function in mammals are conserved in the fly. With the development of ever more sensitive genetic and behavioral tools, utilization of *Drosophila* as a model system has become increasingly prevalent. *Drosophila* models have led, and continue to lead, to advancements in numerous areas of neuroscience. *Drosophila* are an appealing candidate for studying alcohol-related behaviors for a few reasons: face validity, mechanistic validity, and experimental amenability.

### Face Validity

**Figure 1:**
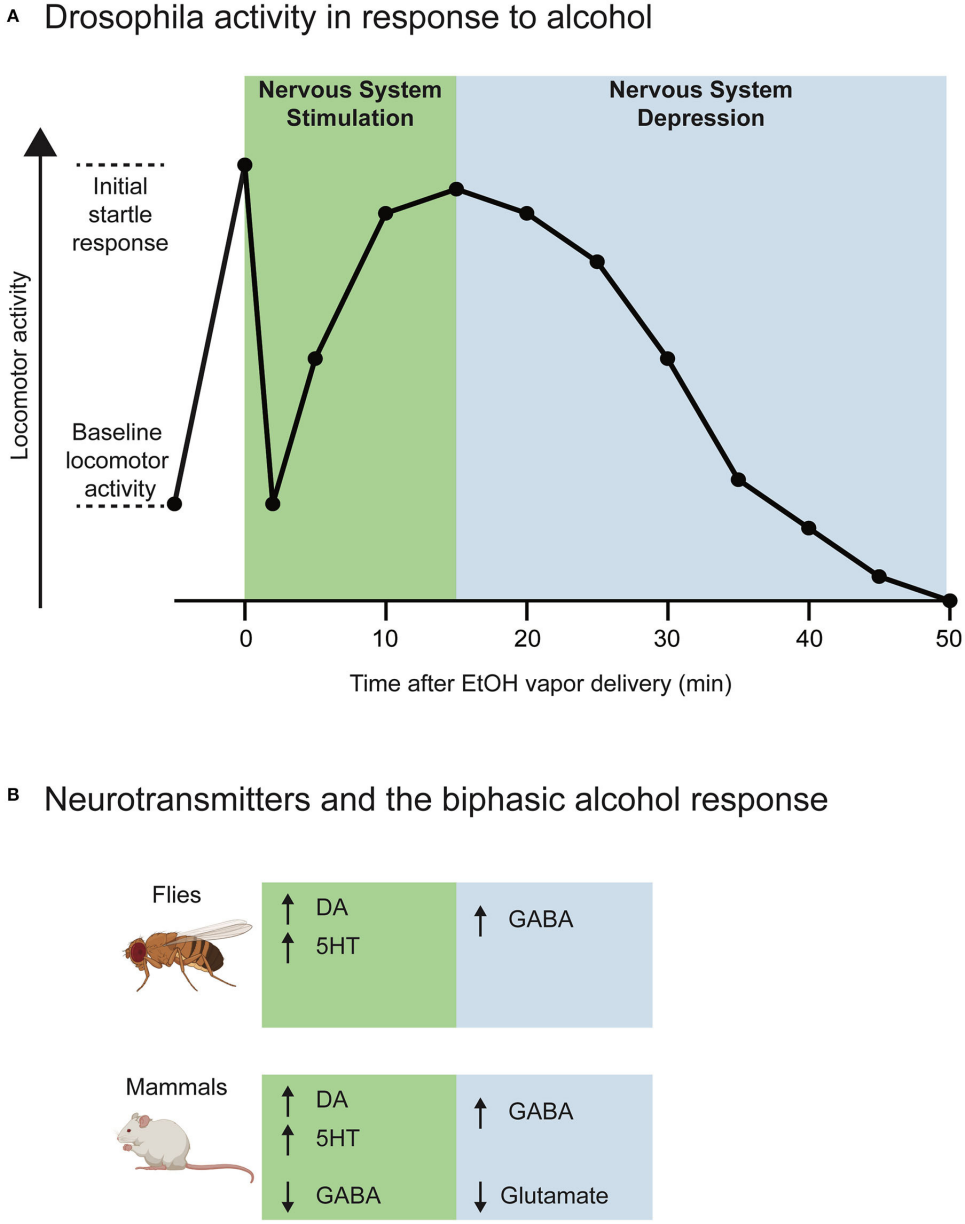
The biphasic alcohol activity response in *Drosophila*, and neurotransmitter involvement in fly and mammalian alcohol responses. **(A)** A sample activity plot shows the change in locomotion of a fly across time following exposure to alcohol vapor. Immediately after ethanol vapor is delivered, the fly has an initial startle response, significantly increasing locomotion from baseline. This startle response quickly drops off and the fly's activity returns to a level close to baseline. As absorption of alcohol takes place, the fly's locomotion gradually increases as nervous system stimulation occurs. Eventually, intoxication peaks, and the fly enters the sedative phase associated with nervous system depression, and activity declines over time until the fly is completely sedated (Bainton et al., 2000; Singh and Heberlein, 2000; Wolf et al., 2002). EtOH = ethanol **(B)** The stimulatory and sedative phases involve distinct neurotransmitter actions. The biphasic alcohol response (nervous system stimulation in green and nervous system depression in blue) is very similar in *Drosophila* and mammals, and some of the same neurotransmitter actions have been implicated in these responses. Arrows indicate increase or decrease in activity for the specified neurotransmitter. See the main text for further details and references.

Face validity describes how much a model “looks like” the disorder being modeled. In our case, face validity is the degree to which *Drosophila* recapitulate alcohol-induced behaviors seen in humans. Accordingly, *Drosophila* show behavioral and neurobiological responses to alcohol that are very consistent with humans and other mammalian species. These include locomotion changes, development of tolerance, learned preference, withdrawal symptoms, and effects on social behavior (Devineni and Heberlein, 2013). One feature of alcohol's neurobiological activity is the biphasic behavioral response: a period of nervous system stimulation followed by a period of nervous system depression. In the stimulatory phase, blood alcohol content rises, and an individual may experience disinhibition, euphoria, and hyperactivity (Fadda and Rossetti, 1998). Later, during the sedative phase, as blood alcohol content peaks, an individual becomes less active, experiencing motor and cognition impairment, and eventually coma and death (Fadda and Rossetti, 1998; Hendler et al., 2013). This biphasic action likely contributes to the development of alcohol dependence. The association of rising blood alcohol content with elevated mood during the stimulatory phase may positively reinforce alcohol drinking (Addicott et al., 2007). The biphasic alcohol response (see Figure 1) is also noted in *Drosophila* (Bainton et al., 2000; Singh and Heberlein, 2000). Upon exposure to ethanol vapor in a video tracking assay, flies show an initial peak of hyperactivity in response to the vapor lasting less than a minute, due to a sensory startle response to ethanol's odor. Following habituation to the odor, flies' locomotion level lessens compared to the startle response. As flies begin to absorb the ethanol and experience intoxication, their locomotion starts to increase again as a consequence of ethanol's pharmacodynamic action on the brain. With further exposure, locomotor activity begins to decline, and sedation takes effect, indicating that flies experience a biphasic ethanol response similar to mammals (Wolf et al., 2002).

**Figure 2:**
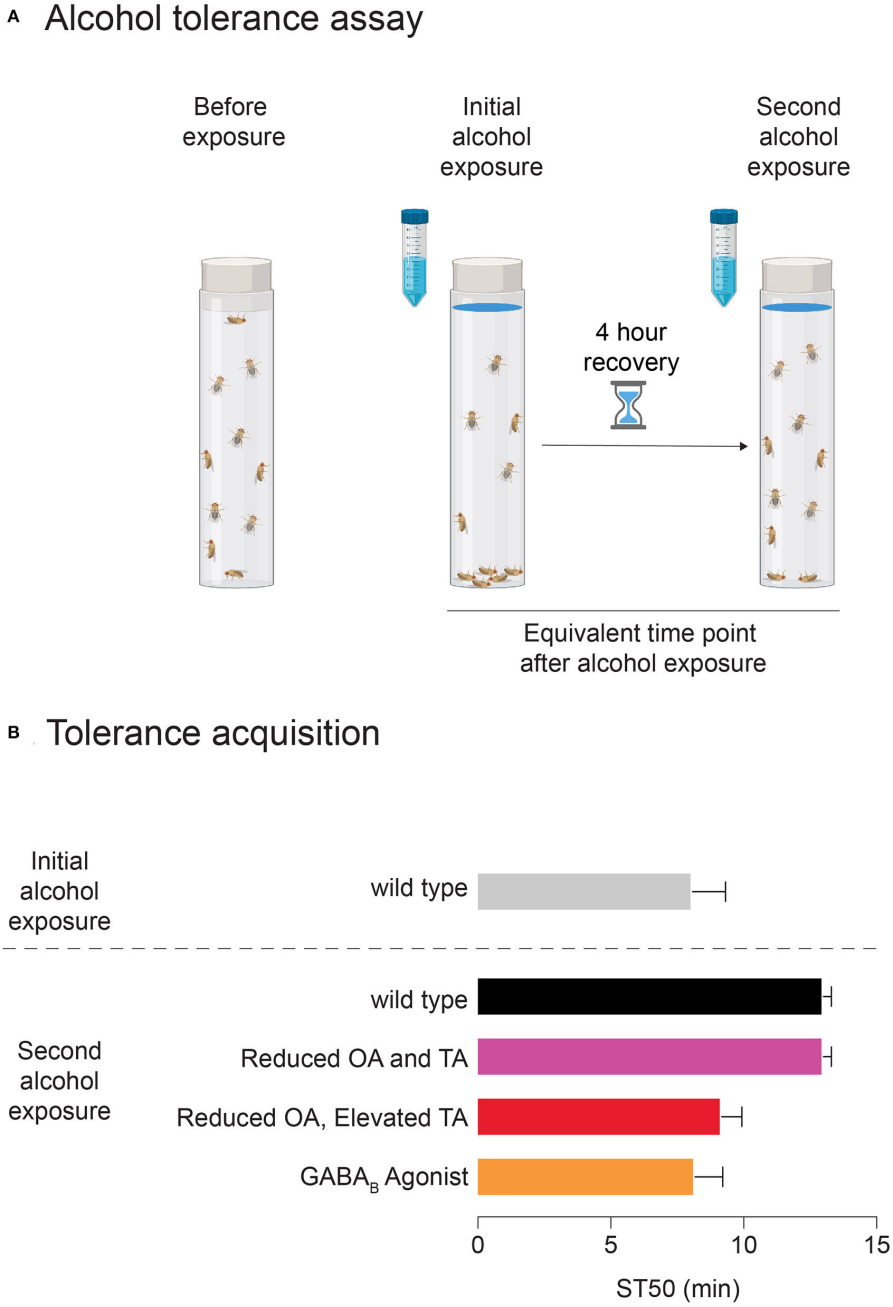
The development of alcohol tolerance in *Drosophila***. (A)** Schematic of alcohol vapor exposure assay. Blue-dyed alcohol is applied to the vial plug, and following the first exposure, flies recover for 4 hours in fresh air. In the second exposure, at the same time point, fewer flies are sedated that in the first exposure, indicating that tolerance has developed. **(B)** Sample data shows ST50 (time it takes for 50% of the flies to become sedated) for a first and second alcohol vapor exposure for wildtype flies and flies with manipulations of different neurotransmitters. These manipulations are either genetic (“Reduced OA and TA” and “Reduced OA, Elevated TA”) or pharmacological via drug feeding (GABA agonist).

*Drosophila* mirror other characteristics of the mammalian alcohol response, including tolerance, withdrawal, and reinforcing properties leading to learned preference for alcohol. Functional tolerance involves adaptations in neuronal activity following exposure to a psychoactive substance, rather than metabolic tolerance, which depends on changes to enzymatic metabolism of ethanol. As functional tolerance develops, a person (or fly) requires increasing amounts of alcohol to become intoxicated in the future (see Figure 2). *Drosophila* demonstrate functional tolerance in as little as 2 h after an initial alcohol exposure (Scholz et al., 2000). Flies and humans also have similarities regarding withdrawal from alcohol. Withdrawal causes a variety of psychological and physiological symptoms, and because drinking alcohol alleviates these symptoms, such attempts to curb withdrawal may contribute to the persistence of AUD (Schuckit, 2009). In *Drosophila* larvae and adults, alcohol withdrawal is associated with neuronal hyperexcitability, which also occurs in humans (Bayard et al., 2004; Cowmeadow et al., 2006; Ghezzi et al., 2014).

**Figure 3:**
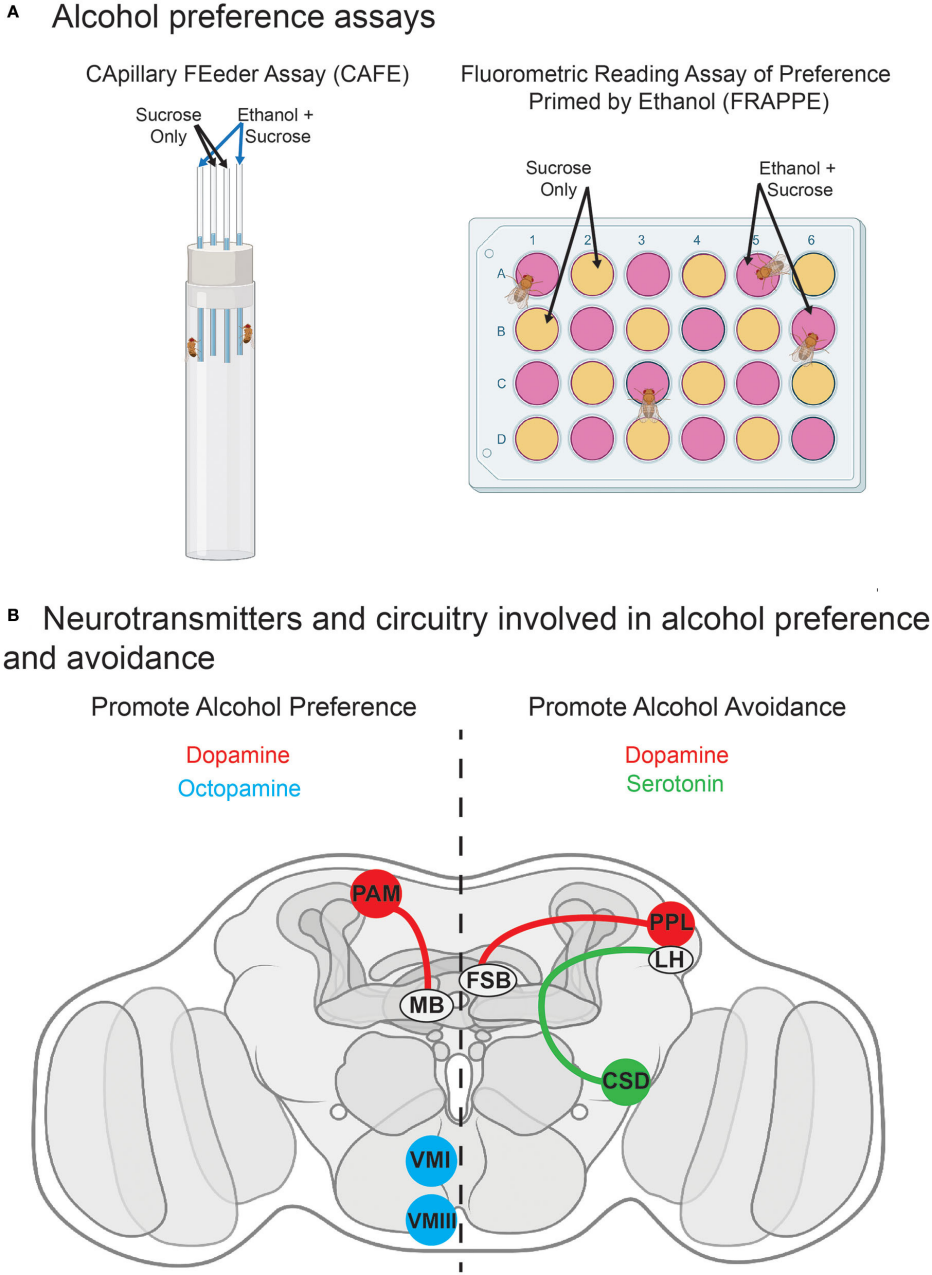
Alcohol consumption preference and related neurotransmitter circuitry in *Drosophila*. **(A)** Schematics of common assays for alcohol preference. The capillary feeder (CAFÉ) and fluorometric reading assay of preference primed by ethanol (FRAPPE) assays are consumption assays in which flies have the choice between two food sources, one with and one without ethanol. **(B)** Known circuitry for neurotransmitters mediating alcohol preference and avoidance. DAergic projections from the PAM cluster to the mushroom body (Ojelade et al., 2019) and OAergic VMI-VMIII neurons (Schneider et al., 2012) promote alcohol preference. DAergic projections from the PPL cluster to the fan-shaped body (Ojelade et al., 2019) and serotonergic signaling in CSD interneurons (Kasture et al., 2019) promote alcohol avoidance. FSB, fan-shaped body; LH, lateral horn; MB, mushroom body. See the text for further details and additional references.

Additionally, like humans, flies can develop a learned preference for alcohol (see Figure 3). Similar to mammals, *Drosophila* do not have an innate preference for alcohol. Upon a first offer of ethanol for consumption, flies are either indifferent or avoidant, depending on the exact presentation parameters (Devineni and Heberlein, 2009; Peru y Colón de Portugal et al., 2014). However, flies develop persistent, experience-dependent preference following exposure to alcohol (Peru y Colón de Portugal et al., 2014). Flies' acquisition of preference for alcohol is a critical component of their usefulness as an animal model. Humans similarly develop alcohol preference that can drive problematic drinking behavior and lead to AUD (Fadda and Rossetti, 1998). In mammals, preference frequently becomes attached to specific contexts or patterns, a phenomenon examined in a conditioned place preference (CPP) assay, wherein a particular environmental context gains attractive qualities after repeated pairing with a drug (Cunningham et al., 2006). Similar behavioral reinforcement occurs in flies, when they acquire preference for an innocuous odor that has been paired with alcohol vapor (Kaun et al., 2011).

### Mechanistic Validity

Mechanistic validity refers to the consistency of neurobiological mechanisms and molecules underlying alcohol response between *Drosophila* and humans. Historically, much of the complexity of studying alcohol lies in its widespread effects throughout the brain. While some drugs, like cocaine, act primarily through a single mechanism (blocking monoamine reuptake into the presynaptic terminal, in cocaine's case) (Hummel and Unterwald, 2002), alcohol's effects on neurotransmission occur in a dose-dependent manner that involves diverse effects across neurotransmitters. Ethanol easily crosses the blood-brain barrier and acts much more globally than other drugs, meaning its mechanisms of action occur quickly and efficiently, contributing to alcohol's propensity for abuse. These diverse mechanisms of alcohol action are consistent from flies to humans.

Many of the genes implicated in mammalian alcohol reactions and human AUD have conserved functions in *Drosophila* (Grotewiel and Bettinger, 2015; Lathen et al., 2020). Some of these involve common molecular pathways for alcohol response, such as cyclic adenosine monophosphate (cAMP) (Moore et al., 1998) and neuropeptide Y (neuropeptide F in flies) (Wen et al., 2005). There is also a high level of conservation in neurotransmitter systems between *Drosophila* and vertebrate species, including humans. While behavioral outcomes may differ, vertebrates and invertebrates share signaling by glutamate, gamma-aminobutyric acid (GABA), acetylcholine (ACh), glycine, dopamine (DA), serotonin (5HT), and numerous neuropeptides (Deng et al., 2019). Although in flies there is no evidence of adrenergic signaling, octopamine (OA), and tyramine (TA) fulfill behavioral roles similar to norepinephrine (NE) and epinephrine in mammals.

In mammals, several neural circuits are involved in behavioral responses to alcohol and the development of AUD [see Abrahao et al. (2017) for a recent review]. For example, in the ventral tegmental area of the mammalian brain, the mesolimbic dopamine pathway is involved in mediating reinforcement and communicating with the nucleus accumbens to drive reward signaling. The fly brain's neuroanatomical structure differs from mammals', but both have circuits implicated in specific behaviors. There are several anatomical regions of interest in the discussion of alcohol-related behaviors. These include the mushroom bodies (MB), a center for associative learning, the antennal lobe (AL), the primary olfactory processing center, and the central complex, which houses the fan-shaped body (FSB), a center of higher integration, and ellipsoid body (EB), a pre-motor structure. Like in mammalian neural circuits, inputs to these brain regions by specific neurotransmitters mediate various alcohol-induced behavioral responses. These are most well-studied for dopamine.

### Experimental Amenability

In *Drosophila*, there are many tools available for genetic manipulations, including forward genetics (going from a phenotype to a causative gene), reverse genetics (going from a targeted mutated gene, using a system like CRISPR/Cas9 mutagenesis, to a phenotype), and genomic approaches (Griffiths et al., 2000). One reverse genetics approach is the GAL4/UAS system (Brand and Perrimon, 1993). This approach involves one transgene carrying GAL4 (a transcriptional activator in yeast), which is under the control of a specific promoter determining the spatial and temporal expression of GAL4. This is combined with a second effector transgene under the control of the upstream activating sequence (UAS), where GAL4 binds. A plethora of distinct GAL4 lines exist, including thousands that drive GAL4 expression in different subsets of neurons (Jenett et al., 2012). The effector transgenes include cDNAs for overexpression, RNAi for gene knockdown, or tools to activate and silence neurons under experimenter control. In conjunction with these genetic tools, *Drosophila's* relatively low cost, ease of maintenance, and short generation time make it amenable to a variety of experimental manipulations.

## Assaying Ethanol-Induced Behaviors

Scientists have studied ethanol responses in *Drosophila* since the 1920s. Early research involved exposing flies to ethanol vapor and measuring time to easily observable behaviors such as sedation or death (Pearl et al., 1929; Crozier et al., 1936). Still today, the basis of many assays lies in similar continuous exposure to ethanol vapor. The benefit of assays involving ethanol vapor is that the exposure time is directly proportional to the flies' level of ethanol absorption, giving researchers more control of the level of intoxication of the flies than in assays that require ingestion. Although humans generally drink alcohol rather than inhaling its vapor, both forms of ethanol exposure result in similar behavioral outcomes. In fact, in recent years, alcohol vaporization has become an increasingly common approach for inducing the development of alcohol dependence in rodent models (Avegno and Gilpin, 2019), suggesting the validity of this approach across organisms.

Several systems exist for exposing flies to alcohol and measuring behavioral output. One of these, the inebriometer, assesses fly postural control following exposure to ethanol vapor (Weber, 1988). The inebriometer evaluates alcohol sensitivity and tolerance. Sensitivity is a measure of the effects of intoxication, typically quantified by locomotor changes leading to sedation. As described previously, tolerance is the development of resistance after an intoxicating dose of alcohol, measured in increased time to intoxication following repeated exposure to alcohol. The inebriometer is a vertical cylindrical tube lined with mesh baffles that slope toward the bottom of the tube. Flies are introduced through the top of the tube, and in the presence of fresh air, they naturally tend to stay at the top of the tube. However, when ethanol diffuses through the tube, the flies lose their ability to hold on to the mesh baffles and eventually fall to the bottom of the tube (Weber, 1988). The inebriometer assesses sensitivity to ethanol sedation by measuring the amount of time it takes for flies to elute through the bottom of the tube (Weber, 1988). Through a process of exposure, elution, recovery, then re-exposure, the inebriometer has also shown that flies develop tolerance to alcohol. As flies develop functional tolerance, they will require more alcohol to become intoxicated. When flies were reintroduced to the inebriometer 4 h after the first exposure (in the meantime fully recovering from the initial intoxication), the mean time of elution through the bottom of the inebriometer in the second exposure increased by ~34% compared to the first exposure, indicating that flies were more resistant to ethanol sedation in the second exposure and that they developed tolerance (Scholz et al., 2000). The contribution of pharmacokinetic changes to this process was ruled out by measuring the ethanol content of prepared fly extracts after exposing naïve and tolerant flies to ethanol vapor. Significantly, the rate of alcohol absorption and metabolism was not significantly different in tolerant flies, suggesting that the development of tolerance is functional rather than metabolic (Scholz et al., 2000).

As described in the previous section, alcohol induces a biphasic behavioral response that impacts activity levels. While the inebriometer essentially indicates whether flies have become sedated or not, other assays can more sensitively quantify locomotor changes. For example, video tracking of walking flies provides a detailed image of locomotion across time. This technique shows that flies have an initial hyperactive startle response to the smell of alcohol, followed by a leveling of the startle response, then gradual increase and decline of activity across time, providing evidence for the biphasic ethanol response in flies (Wolf et al., 2002).

More recently, researchers have developed tools to deliver alcohol to flies in a more translationally relevant way. Although flies will eat alcohol mixed into their food, it has historically been challenging to quantify the amount of food consumed. The capillary feeder (CAFÉ) assay Figure 3A has provided a mechanism to overcome this problem by providing food through a glass microcapillary, allowing for precise measurement of consumption by individuals or groups of flies (Ja et al., 2007). Assays such as this one show that *Drosophila* can develop preference for alcohol, choosing ethanol-containing food over standard food (Devineni and Heberlein, 2009). As described in relation to face validity, learned preference is a key feature of human alcohol use, and its recapitulation in *Drosophila* is a critical component of their fitness as an animal model.

*Drosophila* have a long history of utilization in the study of behavioral responses to alcohol. They have proven especially useful in the discovery and validation of genes affecting alcohol responses. As researchers have developed knowledge of ethanol-related behaviors and tools to assess them, they have asked increasingly complicated questions about the neurobiology underlying these behaviors. The study of specific neurotransmitters is a topic critical to a thorough understanding of behavioral responses to alcohol. As we will discuss in the next section, neurotransmitters are both highly conserved from the mammal to the fly, and they are critically involved in the neurobiological activity of alcohol. With the development of more sensitive genetic tools, the investigation of neurotransmitters has become more attainable.

## Overview of Neurotransmitters

Neurotransmitters can exert excitatory, inhibitory, or modulatory effects. In general, excitatory neurotransmitters increase the likelihood that a neuron will fire an action potential, while inhibitory neurotransmitters decrease the possibility of an action potential firing. Neurotransmitters exert these actions by altering the flow of ions across the cell membrane. Neuromodulators modify the effects of excitatory or inhibitory neurotransmitters and tend to be involved in the slower, longer-lasting activity necessary for higher-order processes.

Despite many conserved similarities, there are some differences between neurotransmitter systems in vertebrates and invertebrates. In mammals, glutamate functions as the primary excitatory neurotransmitter in the CNS, while in *Drosophila*, ACh has this role. Conversely, flies use glutamate at the neuromuscular junction, while in mammals, that neurotransmitter is ACh (Colombo and Francolini, 2019). Although flies do have glutamatergic neurons in the CNS, their role has historically not been well-understood (Liu and Wilson, 2013); but recent advancements will be discussed below. In vertebrates and flies, GABA and glycine both function as inhibitory neurotransmitters (Frenkel et al., 2017), and the two classes of organisms also share many neuromodulators. *Drosophila* neuromodulators include DA, TA, and OA, which come from the common precursor tyrosine (Li et al., 2016b). TA and OA are the functional fly equivalents of mammalian epinephrine and NE, respectively. Both epinephrine and NE are produced from the breakdown of DA, but neither of these chemicals is physiologically relevant for *Drosophila* or other protostomes (Roeder, 2005). 5HT and DA have more known roles in modifying behavior, and these are regulated similarly in vertebrates and invertebrates (Corey et al., 1994; Pörzgen et al., 2001). In flies, neurotransmitters are implicated in a wide variety of behaviors, which are summarized in Table 1. The roles for specific neurotransmitters in alcohol-related behaviors will be discussed in detail later in this review.

**Table 1:** *Drosophila* behaviors associated with each neurotransmitter.

**Neuro-transmitter**	*Drosophila* behaviors *Indicates behaviors impacted by alcohol	**References**
Dopamine	Aggression	Alekseyenko et al., 2013
	Associative learning*	Tully and Quinn, 1985; Riemensperger et al., 2005
	Aversive association*	Honjo and Furukubo-Tokunaga, 2009
	Circadian rhythms	Allada and Chung, 2010
	Locomotion*	Yellman et al., 1997; Pendleton et al., 2002; Kume et al., 2005; Kong et al., 2010; Strausfeld and Hirth, 2013
	Male courtship behavior*	Liu et al., 2008; Hoopfer et al., 2015; Zhang et al., 2019
	Memory removal	Berry et al., 2012
	Multisensory processing	Wolff and Rubin, 2018
	Olfactory learning and memory*	Cognigni et al., 2018
	Reward signaling*	Liu C. et al., 2012; Yamagata et al., 2015
	Salience-based decision making	Zhang et al., 2007
	Sleep and arousal*	Foltenyi et al., 2007; Van Swinderen and Andretic, 2011; Strausfeld and Hirth, 2013
Octopamine	Aggression	Zhou et al., 2008
	Appetitive and aversive associative learning*	Iliadi et al., 2017
	Egg-laying	Monastirioti et al., 1996
	Locomotion*	Sombati and Hoyle, 1984; Saraswati et al., 2004
	Male and female courtship behavior*	Zhou et al., 2012; Rezával et al., 2014
	Odor processing*	Farooqui et al., 2003
	Positive reinforcement for olfactory learning and memory*	Schwaerzel et al., 2003
	Reward*	Hammer, 1993
	Stress response	Hirashima et al., 2000; Chentsova et al., 2002
Tyramine	Flight behavior	Ryglewski et al., 2017
	Locomotion*	Sombati and Hoyle, 1984; Saraswati et al., 2004
	Stress response	Chentsova et al., 2002
	Male courtship behavior*	Huang et al., 2016
Serotonin	Aggression	Alekseyenko et al., 2010
	Associative learning*	Sitaraman et al., 2008
	Circadian rhythms	Yuan et al., 2005
	Depression-like behaviors	Ries et al., 2017
	Hunger and feeding*	Albin et al., 2015; Majeed et al., 2016
	Locomotion*	Silva et al., 2014; Majeed et al., 2016
	Long-term memory formation*	Sitaraman et al., 2008; Scheunemann et al., 2018
	Odor processing*	Ellen and Mercer, 2012
	Sensory perception	Kaneko et al., 2017; Chakraborty et al., 2019
	Sleep*	Liu et al., 2019
GABA	Associative olfactory learning*	Liu et al., 2007
	Labile memory	Pitman et al., 2011
	Locomotion*	Leal and Neckameyer, 2002; Leal et al., 2004
	Sleep length and onset*	Agosto et al., 2008; Chen et al., 2015
	Sleep and memory consolidation*	Haynes et al., 2015
Acetylcholine	Aversive association*	Silva et al., 2015; Bielopolski et al., 2019
	Olfactory learning*	Barnstedt et al., 2016
	Nicotine-induced locomotor changes	King et al., 2011; Fuenzalida–Uribe et al., 2013; Ren et al., 2015
	Sleep promotion*	Aso et al., 2014b
Glutamate	Olfactory habituation	Das et al., 2011
	Olfactory learning and memory*	Xia et al., 2005
	Olfactory response*	Liu and Wilson, 2013
	Sleep regulation*	Guo et al., 2016
	Wake promotion*	Sitaraman et al., 2015; Zimmerman et al., 2017

*Researchers are elucidating which neurotransmitters are involved in regulating the wide behavioral repertoire of Drosophila. Behaviors that could be impacted by or related to alcohol exposure are marked with an asterisk*.

### Alcohol and Neurotransmitters in Mammals

Much of our knowledge about the effects of alcohol on neurotransmitters comes from studies in mammalian models, particularly rodents. Briefly, we will discuss these findings as a point of comparison with *Drosophila*. As mentioned in the introduction, alcohol exerts action by taking advantage of existing biological pathways, necessitating the study of alcohol in the context of known impacts on these pathways and subsequent behavioral alterations. Alcohol's effects on neurotransmission occur in a dose-dependent manner, differentially impacting neurotransmitter systems (Hummel and Unterwald, 2002). Ethanol acts quickly, efficiently, and globally. As discussed in relation to flies' face validity, alcohol's effects are biphasic: initial low doses produce euphoria and hyperactivity, while over time, higher doses depress activity and eventually lead to sedation (Carlsson et al., 1972; Pohorecky, 1977).

The two phases of the alcohol response involve different neurotransmitter systems Figure 1B. At low doses, alcohol acts as a stimulant, causing disinhibition, euphoria, and hyperactivity as blood alcohol content rises (Fadda and Rossetti, 1998). Shortly after ingesting alcohol, mice show a sharp increase in locomotion, attributed to DAergic activation (Carlsson and Lindqvist, 1973). Specifically, these behaviors arise from increased release of DA in the brain's reward system, a mechanism demonstrated in rodents (Yim et al., 1998) as well as humans (Boileau et al., 2003). Due to its involvement in reward processing, DA contributes to both the development and persistence of alcohol dependence (Di Chiara, 1995). In rats, even very small amounts of alcohol administered intravenously increase DA levels in the brain's reward centers and contribute to sustained alcohol self-administration (Lyness and Smith, 1992). Rewarding stimuli are processed via DAergic signaling in the ventral tegmental area (VTA) and nucleus accumbens (NAc). Low doses of alcohol cause dose-dependent activation of DAergic neurons in the rat VTA (Gessa et al., 1985), and alcohol acutely increases synaptic DA levels throughout the reward system, but particularly in the NAc (Di Chiara and Imperato, 1988). Alcohol reduces the activity of GABA in the VTA, thereby disinhibiting DAergic neurons and increasing DAergic activity (Kohl et al., 1998). 5HT is also involved in behavioral regulation, including in brain regions responsible for reward processing, which are implicated in AUD. In humans, 5HT metabolites are more plentiful in blood and urine after drinking alcohol, indicating increased serotonergic transmission, and alcohol consumption increases brain levels of 5HT in animal models (LeMarquand et al., 1994a,b). Additionally, 5HT_1B_ (5HT receptor) knockout mice show less ethanol-induced locomotor impairment, indicative of intoxication, across 11 days of ethanol feeding and testing. Therefore, 5HT may have a role in exacerbating the effects of alcohol and in determining alcohol sensitivity (Crabbe et al., 1996). Serotonergic signaling has particular clinical significance due to the comorbidity of AUD with anxiety and mood disorders, which are often treated with selective serotonin reuptake inhibitor (SSRI) drugs (Gimeno et al., 2017).

As alcohol consumption continues, blood alcohol content peaks, and behaviors associated with CNS depression occur. In the sedative phase, alcohol primarily exerts depressant effects by suppressing excitatory neurotransmission and heightening inhibitory neurotransmission. Alcohol activation of GABA_A_ receptors produces cell hyperpolarization via an influx of chloride ions. Co-administration of ethanol and GABA-mimetic drugs, such as baclofen, enhances the sedative effects of alcohol. Similar experiments with GABA antagonists, such as picrotoxin, reduce alcohol-related incoordination (Martz et al., 1983).

Along with enhancing inhibition, alcohol also suppresses excitation. Beginning in the 1980s, researchers investigated the impact of alcohol on glutamate receptors, showing that even small amounts of alcohol could suppress ion flow through *N*-methyl-*D-*aspartate (NMDA) receptors in cultured rat neurons (Lovinger et al., 1989). Alcohol limits the NMDA-mediated release of neurotransmitters like DA, NE, and ACh, further impairing communication between neurons (Göthert and Fink, 1989; Woodward and Gonzales, 1990). These findings provide a starting point for understanding neurotransmitters' involvement in behavioral responses to alcohol. However, genetic manipulations necessary for greater mechanistic insight are more limited in mammalian models than invertebrates. Therefore, *Drosophila* are an appealing candidate for probing this relationship in greater detail.

### Alcohol and Neurotransmitters in *Drosophila*

*Drosophila* are a useful organism for the study of neurotransmitters because, as described, neurotransmitters are well-conserved from flies to mammals, and they often exert similar effects on behavior. These behavioral effects are particularly useful when considering the effects of alcohol since there is no unique neurobiological pathway for alcohol. However, alcohol has known effects on neurotransmitters that are associated with changes in behavior. See Table 2 for a summary of neurotransmitter roles in alcohol-related behaviors. Additionally, flies have over 40 neuropeptides and signaling hormones, many of which are shared with vertebrate species (Hewes and Taghert, 2001). The best-studied in the context of alcohol is Neuropeptide F (NPF). NPF has a role in *Drosophila* alcohol-related behaviors such as consumption, conditioned preference (Shohat-Ophir et al., 2012; Bozler et al., 2019), and preference for egg-laying in alcohol-containing food (Kacsoh et al., 2013). However, here we focus on small molecule neurotransmitters.

**Table 2:** Role of neurotransmitters in alcohol-related phenotypes in *Drosophila*.

**Neuro-transmitter**	**Part of pathway**	**Activation (+)/Blockage (–) of function**	**Pharm. (P) or genetic (G) manipulation**	**Alcohol-related phenotype(s)**	**Reference**
Dopamine	N/A	N/A	N/A	Alcohol potentiates global DA release	Ojelade et al., 2019
	Tyrosine hydroxylase	–	P	Decreased acute hyperactivation	Bainton et al., 2000
	DA neuron synaptic transmission	–	G, P	Decreased acute hyperactivation	Kong et al., 2010
				Increased naïve preference	Ojelade et al., 2019
		–	G	Decreased disinhibition	Lee et al., 2008
		+	G, P	Increased naïve aversion	Ojelade et al., 2019
	DAT	–	G	Decreased acute hyperactivation	Kong et al., 2010
	Central complex neuronal activity	+	G	Increased acute hyperactivation	Kong et al., 2010
	Dop1R1 receptor	–	G	Decreased acute hyperactivation	Kong et al., 2010
				No change in rapid tolerance	Kong et al., 2010
				Increased naïve preference	Ojelade et al., 2019
	Both D1-like receptors	–	G	No change in sensitization to disinhibition	Aranda et al., 2017
	DopEcR	–	G	Acute sedation resistance	Petruccelli et al., 2016
				Increased acute hyperactivation	Petruccelli et al., 2016
				Decreased sensitization to disinhibition	Aranda et al., 2017
	PPL DAergic neurons projecting to FSB	–	G	Decreased naïve aversion	Ojelade et al., 2019
	DA neurons	–	G	Decreased conditioned preference	Kaun et al., 2011
Octopamine and tyramine	Tyrosine decarboxylase	–	G	Decreased acute hyperactivation	Scholz, 2005
				Acute sedation resistance	Chen et al., 2013
	Tyramine beta-hydroxylase	–	G	Increased acute hyperactivation	Scholz, 2005
				Decreased rapid tolerance	Scholz et al., 2000; Scholz, 2005
				Decreased startle response	Scholz, 2005
				Decreased olfactory preference	Schneider et al., 2012
				Decreased olfactory attraction	Claßen and Scholz, 2018
		+	G	Decreased sensitivity	Chen et al., 2013
	Global OA levels	+	P	No change in sensitivity	Chen et al., 2013
				Increased olfactory attraction	Claßen and Scholz, 2018
	OA neurons	+	G	Induced olfactory preference for EtOH	Schneider et al., 2012
	OA receptor	–	G, P	Decreased olfactory attraction	Claßen and Scholz, 2018
	TA receptor	–	G	No change in acute sedation	Scholz, 2005
			P	Decreased sensitivity	Chen et al., 2013
Serotonin	Global 5HT levels	+	P	Decreased olfactory preference	Xu et al., 2016
	SerT	–	G	Decreased olfactory attraction	Xu et al., 2016
				Decreased olfactory preference	Kasture et al., 2019
		–	P	No change in sensitivity	Chen et al., 2010
	SerT in CSD neurons	+	G	Increased olfactory aversion	Kasture et al., 2019
	5-HTP	+	P	Increased sensitivity	Chen et al., 2010
	PKC53E in 5HT neurons	–	G	Reduced activity of 5HT neurons	Chen et al., 2010
				Decreased sensitivity	Chen et al., 2010
GABA	GABA_B_R	+	P	Increased sensitivity	Ranson et al., 2020
				Increased chronic tolerance	Ranson et al., 2020
				Decreased rapid tolerance	Dzitoyeva et al., 2003
		–	P	Decreased sensitivity	Ranson et al., 2020
				Decreased alcohol-induced motor impairment	Dzitoyeva et al., 2003
Glutamate	DAergic projections to glutamatergic MBONs	N/A	N/A	Consolidation of alcohol-related memories	Scaplen et al., 2020

*For each neurotransmitter, the different manipulations reported are listed (part of the neurotransmitter pathway, whether that component of the pathway is being activated or blocked, and whether the manipulation is pharmacological or genetic) together with their behavioral output in the presence of alcohol. In 2 cases (“N/A” in columns 2–3), no manipulation is indicated and a general behavioral response to alcohol is shown. See the main text for detailed descriptions of the listed behaviors. EtOH = ethanol*.

## Dopamine

In *Drosophila*, DAergic neurons are distributed throughout the CNS (Budnik and White, 1988) but comprise only about 250 of the ~100,000 neurons in the fly brain (Mao and Davis, 2009; Aso et al., 2010; Zheng et al., 2018). Despite the relatively small number of DA neurons in the adult brain, DA is involved in many *Drosophila* behaviors (see Table 1 for a summary). Recent work indicates that many DAergic neurons have distinct functions depending on the specific circuitry in which they are involved (e.g., Azanchi et al., 2013; Ojelade et al., 2019). These recent developments paint an optimistic picture for future advancements regarding neurotransmitters that are currently poorly understood, such as glutamate, GABA, and ACh.

The fly brain does not structurally resemble the mammalian brain, although *Drosophila* have neural circuits fulfilling roles similar to those of the vertebrate brain. Some anatomical regions of interest to the discussion of DA are the central complex, which houses the fan-shaped body (FSB) and ellipsoid body (EB), and the mushroom bodies (MB). DAergic neurons reside in 10 distinct clusters per hemisphere (Xie et al., 2018). Each cluster has stereotyped projections to other brain regions and distinct roles in behavior (Nässel and Elekes, 1992; Mao and Davis, 2009), which we will discuss below.

### Dopamine Synthesis, Action, and Metabolism

DA is produced by the metabolism of essential amino acid phenylalanine or its metabolite, non-essential amino acid tyrosine. Dietary ingestion is the primary source of both phenylalanine and tyrosine. Tyrosine is converted by tyrosine hydroxylase (TH) to L-DOPA, which is then converted to DA by dopamine decarboxylase (Cole et al., 2005). There are two classes of DA receptors, classified due to their similarities to mammalian DA receptors: D1-like and D2-like. There are two D1-like receptors, Dop1R1, which signals via Gαs to stimulate cAMP production, and Dop1R2, which couples to Gαq to increase cytosolic calcium (Handler et al., 2019). The D2-like receptor, Dop2R, inhibits the adenylyl cyclase/cAMP pathway (Scholz-Kornehl and Schwärzel, 2016). Both classes are G protein-coupled receptors (GPCRs) with seven transmembrane domains (Karam et al., 2020). Flies also have DopEcR, a G protein-coupled DA/ecdysteroid receptor that can be activated by either DA or the insect hormone ecdysone (Srivastava et al., 2005). Much of the structure of these receptors is conserved between vertebrates and *Drosophila* (Karam et al., 2020). After the presynaptic neuron releases DA into the synapse and DAergic action occurs, the dopamine transporter (DAT) takes DA back up into the neuron.

### Dopamine and Ethanol in *Drosophila*

DA has numerous important functions in *Drosophila* behavior, in part due to the widespread projection of DAergic neurons throughout the brain. Circuits responsible for the alcohol response involve anatomical regions such as the MB and central complex, specifically the EB. There is an extensive body of research on DA's roles in fly behavior, so we will focus here on the behaviors and neural circuitry most relevant for alcohol, namely locomotion, which involves the central complex, and learning and memory, punishment, and reward, which involve the MB. DAergic inputs to the central complex mediate motor activity and sleep (Strausfeld and Hirth, 2013), multisensory processing (Wolff and Rubin, 2018), and social behaviors like aggression (Alekseyenko et al., 2013). See Table 1 for a summary of DA roles in fly behavior.

Like in mammals, alcohol impacts the *Drosophila* DAergic system (Bainton et al., 2000), and many DA-related behaviors are linked to and affected by alcohol. Alcohol affects several DA-mediated behaviors in *Drosophila*, such as locomotion, sedation, and reward. Additionally, DA has an important role in flies' preference for laying eggs in ethanol-containing food. Subsets of competing DA neurons enhance or inhibit this preference (Azanchi et al., 2013), possibly suggesting a DAergic role for flies' innate attraction to alcohol's odor at low concentrations (Ogueta et al., 2010). Significantly, alcohol potentiates the release of DA in the fly brain, which may explain the noted enhancement of locomotion and reinforcing behavioral effects following ethanol exposure (Ojelade et al., 2019).

#### Locomotion and Sedation: Ellipsoid Body

In general, increased DAergic signaling is associated with increased locomotion, while decreased DAergic signaling is associated with reduced locomotion. In decapitated flies with an exposed nerve cord, application of DA stimulated locomotion and hindleg grooming, while application of a DA antagonist significantly blocked this behavior (Yellman et al., 1997). DA signaling specifically to the EB regulates locomotion (Kong et al., 2010). In studies with live flies, a DAT mutation also increased locomotive excitability and prolonged response to a mechanical stimulus, suggesting a critical role of DA in the regulation of movement and arousal (Kume et al., 2005). Loss-of-function mutations of the Dop1R DA receptor has been further implicated in elevating startle-induced arousal (the focus for Kume et al., 2005) while decreasing arousal from sleep, suggesting a role for DA activity in independently modulating different forms of arousal (Lebestky et al., 2009).

The role of DA in ethanol-related alterations to locomotion is increasingly well-known in flies. When placed in narrow tubes, flies show a basal activity level that increased for 7–10 min upon exposure to ethanol vapor (Bainton et al., 2000), consistent with the biphasic ethanol response. Certain DAergic perturbations reduce this alcohol-related locomotor activity. For example, in flies fed 3-iodotyrosine (3IY; a competitive antagonist of TH that reduces global DA levels), the shape of the biphasic locomotor response curve was similar to control flies, but the amount of locomotion was significantly blunted, which could be reversed by feeding L-DOPA (Bainton et al., 2000). Therefore, DA has a role in modulating ethanol-induced hyperactivity in flies, like in mammals. Additionally, the tetanus toxin light chain (TeTx), which blocks synaptic transmission (Sweeny et al., 1995), was expressed in a subset of EB-projecting DA neurons using GAL4-UAS (Kong et al., 2010). In TeTx-expressing flies, locomotor activity was significantly reduced compared to controls. However, coordination appeared normal, and the odor-induced startle response to the introduction of ethanol vapor was not affected (Kong et al., 2010). These findings highlight the specificity of *Drosophila* neural circuitry for the modification of unique behaviors. Even within a single neurotransmitter system, individual neurons and neuron subsets have distinct functions depending on the circuitry in which they are involved.

Studies have also attempted to unravel if specific DAergic neurons and receptors are involved in locomotive responses to acute ethanol exposure. In transgenic lines expressing dopamine decarboxylase using GAL4 drivers for subsets of TH-containing neurons, specific DA neurons in the PPM3 cluster and target neurons in the central complex EB promote locomotion (Kong et al., 2010). Additionally, the fly D1-like receptor, Dop1R1, is required for locomotive activation in response to ethanol. None of these neurons was necessary for the olfactory startle response to alcohol, suggesting that alcohol acts on PPM3 DA neurons that signal to the EB through Dop1R1 to evoke a motor response (Kong et al., 2010).

The sedative effects of alcohol have also been an area of investigation concerning DA. Investigations focused on DopEcR showed that flies with mutations on this receptor took over an hour longer to become sedated than control flies. However, DA was not relevant for the DopEcR activation that promoted this particular behavior. The process is likely mediated by ecdysone, as ecdysone-fed flies overexpressing DopEcR were resistant to alcohol sedation (Petruccelli et al., 2016). Although DA does not impact ethanol-induced sedation via DopEcR, DA may act through DopEcR to affect other behaviors. For example, DA action on DopEcR may oppose the ethanol-induced hyperactivity mediated by the two D1-like receptors. Indeed, *DopEcR* mutants show an elevated hyperactive alcohol response, suggesting that wildtype DopEcR is involved in minimizing Dop1R-mediated hyperactivity in response to alcohol (Petruccelli et al., 2016).

#### Associative Learning and Preference: Mushroom Body

DA is necessary for complex behaviors like learning and memory. In *Drosophila*, blocking DA inhibits the acquisition of aversive memories (Honjo and Furukubo-Tokunaga, 2009), and flies lacking the Dop1R2 receptor, highly expressed in the MBs (Han et al., 1996), have impaired removal of memories (Berry et al., 2012). Additionally, DAergic inputs to the MB are vital for olfactory learning and memory (Cognigni et al., 2018), and DAergic projections from the protocerebral anterior medial (PAM) cluster to the MB are involved in reward signaling (Liu C. et al., 2012). Subsets of DA neurons are also involved in reward signaling for short vs. long-term memory (Yamagata et al., 2015). Alterations to the brain's reward pathways are a critical feature of addiction.

DA has a multifaceted role in mediating alcohol-induced behaviors; it influences both reinforcing and aversive alcohol responses (see Figure 3B). Flies are innately indifferent or averse to ingesting alcohol. However, after alcohol exposure, this turns into experience-dependent preference (Peru y Colón de Portugal et al., 2014). DA is involved in both naïve aversion and conditioned preference. Conditioned alcohol preference is the associative learning process by which a fly learns to correlate ethanol with an attractive cue. Although DA was once thought to be involved only in the retrieval of conditioned preference and not acquisition (Kaun et al., 2011), recent evidence suggests a role for DA in preference acquisition (Ojelade et al., 2019). The PAM cluster of DAergic neurons is involved in appetitive olfactory conditioning, and DAergic signaling in these neurons is necessary for experience-dependent alcohol preference (Ojelade et al., 2019). Specifically, PAM DA neuron projections to the MB were necessary for the acquisition of alcohol preference, which is further supported by evidence that knocking down the Dop1R1 receptor in the MB impairs the development of preference (Ojelade et al., 2019). Additionally, recent evidence clarifies the role of DA in the consolidation and retrieval of preference. DAergic activity inhibits specific mushroom body output neurons (MBONs) involved in a circuit for the consolidation of alcohol-related memories. This circuit also converges on the FSB. This inhibition may permit the consolidation of alcohol preference (Scaplen et al., 2020). It is clear that DA plays a dynamic role in the behavioral response to alcohol, and many of these findings have come about in recent years due to advancements in tools for examining the DAergic system. These outcomes are compelling in considering other, less-explored *Drosophila* neurotransmitters, as similar innovations for these neurotransmitters are likely forthcoming.

#### Acute Aversion to Alcohol: Fan-Shaped Body

As mentioned in the previous section, naïve flies initially show indifference or aversion to alcohol consumption. In the CAFÉ assay, the first preference measurement is generally after 24 h and shows indifference (Devineni and Heberlein, 2009; Xu et al., 2012). However, Butts et al. (2019) have shown that some flies can acquire preference in <12 h. Indeed, in preference assays that do not use capillaries to offer the food (Park et al., 2018) or that are shorter in duration (Peru y Colón de Portugal et al., 2014; Butts et al., 2019), flies show initial aversion to alcohol. This was further examined by pharmacologically or genetically manipulating DA, showing that flies with increased DA levels have enhanced naïve alcohol aversion. In contrast, flies with decreased DA levels have naïve alcohol preference (Ojelade et al., 2019). These findings indicate that DA is critical for flies' aversion to alcohol. Regarding circuitry, a pair of DAergic protocerebral posterior lateral (PPL) neurons specifically mediates acute aversion: silencing these neurons abolishes aversion Figure 3B (Ojelade et al., 2019). The PPL cluster of DA neurons mostly projects to the MB (Aso et al., 2014a) to mediate punishment (Handler et al., 2019) and is activated by aversive stimuli (Mao and Davis, 2009). However, one bilateral PPL neuron projects to the FSB (Liu Q. et al., 2012), and this projection mediates the acute aversion to alcohol (Ojelade et al., 2019). The FSB is therefore emerging as a higher center of integration, where output to learned alcohol responses from the MB (Scaplen et al., 2020) merge with acute sensory processing, and here, alcohol aversion (Ojelade et al., 2019). Similar integrative processes have been found for aversive sensory responses and conditioning by electric shock (Hu et al., 2018).

## Octopamine and Tyramine

In *Drosophila*, OA (the NE homolog) and TA (the epinephrine homolog) are expressed in over 100 neurons (Selcho et al., 2014). OA-immunoreactive neurons, which necessarily contain TA, reside in discrete clusters throughout the fly brain (Sinakevitch and Strausfeld, 2006). Although OA is a metabolite of TA, TA has only recently become an independent target of investigations. OA and TA have often historically been explored together by manipulating metabolic steps upstream of TA. OA has many well-characterized independent impacts on insect physiology and behavior, but these roles are less well-defined for TA (Pauls et al., 2018). It was long thought that while DA mediates the formation of aversive memories, OA has a specific role in appetitive memories (Schwaerzel et al., 2003). However, newer evidence suggests that OA is required for both appetitive and aversive learning and, therefore, associative learning in general (Iliadi et al., 2017).

### Octopamine and Tyramine Synthesis, Action, and Metabolism

Like DA, TA and OA are produced by the metabolism of essential amino acid phenylalanine or its metabolite, non-essential amino acid tyrosine, both found in food. Tyrosine is converted to TA by tyrosine decarboxylase (Tdc), and TA is converted to OA by tyramine beta-hydroxylase (Tbh). Manipulating Tbh concentration alters the OA/TA equilibrium: *Tbh-*null flies have increased TA levels and decreased OA levels (Monastirioti et al., 1996). There are 4 GPCRs for OA and 3 for TA. All seven types show high expression in the brain (El-Kholy et al., 2015). Based on parallels with the vertebrate adrenergic system, fly OA receptor classifications include α-adrenergic-like, β-adrenergic-like, and OA/TA or TA receptors (Evans and Maqueira, 2005). These receptors exert a variety of effects. Activation of the α-adrenergic-like receptor leads to elevation of calcium ions, and activation of the three β-adrenergic-like receptors increases intracellular cAMP levels (Balfanz et al., 2005; Maqueira et al., 2005). Interestingly, though researchers have described a specific OA transporter in many insects, one has not been identified in *Drosophila*. OA reuptake in flies may occur via DAT, although this requires further investigation (Arancibia et al., 2019).

### Tyramine/Octopamine and Ethanol in *Drosophila*

TA and OA influence a wide variety of behaviors. Both neurotransmitters were initially of interest to researchers because they are critical for insect physiological processes like modulation of organs and muscles, and since vertebrates lack receptors for both, they provided a potential target for insecticides (Roeder, 2005). Although TA was historically thought to function primarily as a precursor of OA and exert few of its own effects, the presence of TA-activated GPCRs suggests that it may function independently as a neurotransmitter (Borowsky et al., 2001), and TA has been independently implicated in some behaviors. See Table 1 for a summary of OA and TA roles in fly behavior.

Although research has historically focused more on DA, OA, and TA are also involved in behavioral responses to alcohol. OA and TA are implicated in ethanol-related behaviors such as locomotion, sensitivity, tolerance, preference, and olfactory attraction. Investigations often explore TA and OA in conjunction due to their common precursor, and it is not clear whether there are distinct TAergic and OAergic neurons.

#### Locomotion, Sensitivity, and Tolerance

In *Drosophila* larvae, flies with elevated TA and low OA levels had reduced locomotion compared to wildtype, and flies with reduced levels of both OA and TA showed less severe locomotor impairment (Saraswati et al., 2004). Thus, OA and TA exert opposing effects on larval locomotion, and a balance between both is necessary for normal behavior. As described for DA, in an experiment in which amines were applied to the exposed nerve cord of decapitated flies, OA stimulated hindleg grooming and strong locomotion (Yellman et al., 1997), suggesting an important role for OA and TA in mediating locomotion.

Various genetic mutations impacting the OAergic and TAergic systems are known to have roles in modulating alcohol sensitivity and tolerance, and flies with these mutations are useful for unraveling the impacts of TA and OA on alcohol-related behaviors. Some of these mutations impact synthesis enzymes. For example, in a mutant called *inactive* (*iav*) Tdc activity is reduced, causing reduced TA and OA levels (Chentsova et al., 2002), while *Tbh-*null flies have increased TA levels and decreased OA levels (Monastirioti et al., 1996). Upon first ethanol exposure, *iav* flies' locomotion is reduced compared to controls, while *Tbh* mutants show more locomotion than controls (Scholz, 2005). This suggests opposing roles of TA and OA in regulating the locomotive response to alcohol. In regard to tolerance, mutant *iav* flies are sensitive to sedation in the first alcohol exposure, but they develop tolerance and are less sensitive during the second exposure (Scholz, 2005; see Figure 2). Conversely, *Tbh* mutants showed normal alcohol sensitivity in the first exposure but developed less tolerance during the second exposure (Scholz, 2005). To further explore the role of OA in the development of tolerance, *Tbh-*null flies were tested in the inebriometer. While sensitivity to alcohol did not change, 4 h after the initial exposure, *Tbh* flies showed 50–60% less tolerance than controls (Scholz et al., 2000). This effect was not reversed by 2 days of TA-feeding, suggesting that the effect on tolerance development was due to OA and not elevated TA (Scholz et al., 2000).

Later work on the role of TA in ethanol sensitivity identified the *Bacchus* (*Bacc*) gene. While the molecular function of this gene is not well-understood, a loss-of-function mutation of *Bacc* reduced alcohol sensitivity, likely via heightened Tbh activity converting more TA to OA (Chen et al., 2013). In reducing Tbh activity or orally administering TA, *Bacc* mutant flies show normal ethanol sensitivity (Chen et al., 2013). Additionally, in a GAL4 line in which both OA and TA neurotransmission were blocked, flies were significantly resistant to ethanol sedation compared to controls. This phenotype was restored when flies were fed TA or TA plus OA but not OA alone, indicating that *Bacc* does not impact ethanol sensitivity via increased OA activity (Chen et al., 2013). These findings suggest that TA has independent involvement in regulating ethanol response.

#### Olfactory Ethanol Attraction

OA has a role in odor processing (Farooqui et al., 2003), extending to ethanol. Based on the theorized OAergic signaling requirement for the positive association of an odor and stimulus (Schwaerzel et al., 2003), Schneider et al. examined OA's role in olfactory ethanol preference (Schneider et al., 2012). In *Tbh* mutants, flies did not show olfactory preference, a phenotype restored by expressing a Tbh cDNA with a Tdc-GAL4 driver line (Schneider et al., 2012). Researchers also assessed Tbh expression patterns to find OAergic neurons and identified 26 neurons specifically involved in olfactory ethanol preference (Schneider et al., 2012; see Figure 3). Optogenetic targeting of these neurons determined that activation of OAergic neurons is sufficient for inducing preference and that previously noted alcohol preference in response to OA supplementation in *Tbh* mutants was not simply the result of increased neuronal activity (Schneider et al., 2012).

OA also has a critical role in determining behavior via its role in biasing the fly's decision toward food odors. Pharmacologically increasing OAergic signaling increases ethanol attraction, while blocking OA receptors reduces it (Claßen and Scholz, 2018). *Tbh* mutant flies do not initially show ethanol attraction, but it is rescued upon feeding the flies OA or OA receptor agonists. Convergently, feeding wildtype flies epinastine, an OA receptor antagonist, impairs ethanol attraction similarly to *Tbh* mutants (Claßen and Scholz, 2018). Therefore, OA is required for olfactory ethanol attraction. TA has also been investigated in conjunction with OA to understand olfactory attraction. TA-fed wildtype flies showed a slight but insignificant reduction in attraction to ethanol. However, in *Tbh* mutant flies lacking OA, TA feeding significantly induced ethanol attraction (Claßen and Scholz, 2018). It is possible that TA can act as an agonist for OA receptors at high levels or that elevated activation of TA receptors may induce ethanol attraction. Both TA and OA are likely involved in olfactory attraction to ethanol (Claßen and Scholz, 2018).

## Serotonin

The serotonin system in *Drosophila* exerts significant behavioral effects despite the very small number (~80) of serotonergic neurons in the fly brain. These neurons reside in several clusters (Sitaraman et al., 2008). Genetic approaches have shown that regulation of behaviors can stem from individual serotonergic neurons within clusters (Pooryasin and Fiala, 2015). 5HT exerts behavioral and physiological effects on processes such as hunger (Albin et al., 2015), sleep (Liu et al., 2019), and sensory perception (Chakraborty et al., 2019).

### Serotonin Synthesis, Action, and Metabolism

Unlike the previously discussed neurotransmitters, 5HT does not originate from the amino acid tyrosine, but tryptophan. The precursor tryptophan, absorbed in the diet, is converted to 5-hydroxytryptophan (5-HTP) by tryptophan hydroxylase. Then, aromatic amino acid decarboxylase converts 5-HTP to 5-hydroxytryptamine (otherwise known as serotonin or 5HT) (Coleman and Neckameyer, 2005). Flies have five different G protein-coupled 5HT receptors. 5-HT1A, 5-HT1B, and 5-HT7 are all coupled to the cAMP signaling cascade, while 5-HT2A and 5-HT2B activation lead to Ca^2+^ signaling (Blenau et al., 2017). Some of these receptor subtypes are involved in specific outcomes, like the role of the 5-HT2B receptor in minimizing anxiety-like behaviors (Mohammad et al., 2016). 5HT is removed from the synapse via reuptake by the *Drosophila* serotonin transporter (SerT) (Demchyshyn et al., 1994). SerT colocalizes with 5HT neurons throughout the brain (Giang et al., 2011), and its mutations provide a useful tool for investigating phenotypic outcomes of serotonergic signaling.

### Serotonin and Ethanol in *Drosophila*

5HT affects numerous behaviors in *Drosophila*, and manipulation of the fly serotonergic system has recapitulated symptoms of neuropsychiatric disorders like depression and anxiety (Ries et al., 2017). Importantly for consideration of alcohol-related behaviors, 5HT is critical for memory formation in *Drosophila* (Sitaraman et al., 2008). Memory performance worsened by genetically blocking serotonergic neurotransmission during a task for learned avoidance of high temperatures. A similar result was noted upon the pharmacological blockage of 5HT (Sitaraman et al., 2008). See Table 1 for a summary of 5HT roles in fly behavior.

Although historically not the subject of intense research efforts, new evidence increasingly supports a role for 5HT in *Drosophila's* ethanol-related behaviors. These behaviors include olfactory attraction, preference, and sensitivity.

#### Olfactory Attraction

As we discussed in the DA section, *Drosophila* do not have a naïve preference for consuming alcohol. Although there is evidence that they are innately attracted to its odor at low concentrations (Ogueta et al., 2010), it is not clear what the exact role for this attraction is in driving alcohol self-administration. 5HT is involved in odor processing (Ellen and Mercer, 2012), which has made it an appealing candidate for investigating ethanol attraction. In a two-choice assay between a food source with or without ethanol, flies with pharmacologically increased 5HT levels showed significant loss of preference for alcohol's odor (Xu et al., 2016). Also, genetically rendering SerT non-functional, thereby increasing 5HT in the synaptic cleft and prolonging serotonergic signaling, reduced olfactory ethanol attraction. Four serotonergic neurons are implicated in this inhibition (Xu et al., 2016). These researchers went on to explore two neurons distinct from the previously identified four: the contralaterally-projecting, serotonin-immunoreactive deutocerebral (CSD) neurons. The CSD neurons counteract the inhibition of the other four serotonergic neurons (Xu et al., 2016), and they are the only serotonergic neurons innervating the antennal lobes (AL), the fly brain equivalent of the olfactory bulbs (Xu et al., 2016). These are involved with odor detection (Roy et al., 2007). In prolonged exposure to an odor, CSD neurons counteract the inhibition of olfactory attraction by the four previously identified serotonergic neurons and enhance olfactory input via 5HT (Xu et al., 2016). Therefore, 5HT's role in olfactory attraction to ethanol is multifaceted: 5HT generally functions to inhibit olfactory attraction, but the CSD neurons overrule this inhibition in the prolonged presence of an odor (Xu et al., 2016).

In another study of olfactory alcohol preference, researchers generated flies with a non-functional SerT and then placed the flies in a two-choice odor trap with one trap containing ethanol. Flies with a disrupted SerT showed a lower preference for alcohol than wildtype flies, but both groups showed a higher preference for the trap containing ethanol than the one without (Kasture et al., 2019). These effects also have intracellular location-specific characteristics. Restoring SerT in the global mutant in previously described CSD interneurons resulted in olfactory alcohol aversion while restoring SerT function only in the soma and dendrites rescued normal attraction (Kasture et al., 2019; see Figure 3). These findings suggest that 5HT transport exerts unique ethanol-related behavioral effects in the somatodendrities vs. axons (Kasture et al., 2019).

#### Locomotion, Sensitivity, and Sedation

As we have discussed throughout this paper, locomotion is one behavior that is impacted by alcohol. In *Drosophila*, increased 5HT is associated with reduced locomotion. Larvae treated with drugs that increase 5HT signaling [such as fluoxetine and 3,4-Methylenedioxymethamphetamine (MDMA)] decreased their locomotion. Treating larvae with drugs that reduce serotonergic signaling reversed this effect (Silva et al., 2014). This is important in the context of alcohol since locomotion is a behavioral marker for alcohol sensitivity.

Serotonergic signaling is also linked to protein kinase C (PKC), which several studies have shown is involved with alcohol sensitivity (Newton and Ron, 2007). Chen et al. showed that PKC positively regulates 5HT activity to influence ethanol sensitivity. Inhibition of one PKC subtype (PKC53E) in serotonergic neurons reduced the activity of 5HT neurons and reduced sensitivity to ethanol (measured as time to sedation) (Chen et al., 2010). Upon feeding flies an SSRI, ethanol sensitivity was restored to normal, suggesting that PKC53E deficiency influences alcohol-related behaviors via depletion of synaptic 5HT (Chen et al., 2010). 5HT may also be involved in the relationship between diet and ethanol sedation. In general, a high-yeast diet increases 5HT levels in the brain (Ro et al., 2016) and increases flies' resistance to alcohol sedation (Schmitt et al., 2020). Serotonergic neurons can block the sedation resistance caused by a high-yeast diet (Schmitt et al., 2020), suggesting a role for 5HT in mediating the link between diet and ethanol-related behaviors.

In the last several years, there has been an increase in the number of studies investigating the *Drosophila* serotonergic system. However, few of these focus specifically on the role of 5HT in the mediation of ethanol-related behaviors. As genetic and behavioral tools continue to advance, roles for 5HT in behavioral outcomes of alcohol use will continue to be uncovered. An extensive body of research in mammals suggests that increases in serotonergic signaling are associated with decreased alcohol use and vice versa. Additionally, alcohol may elevate 5HT activity to activate DAergic neurons and the reward system (LeMarquand et al., 1994b). Since the *Drosophila* DAergic system is also implicated in the behavioral response to alcohol, flies, and mammals could potentially share mechanisms by which the serotonergic system mediates ethanol-related behaviors.

## GABA

In vertebrates and invertebrates alike, GABA functions as the major inhibitory neurotransmitter. In *Drosophila*, although it does not appear at detectable levels until relatively late in development, GABA is distributed throughout the nervous system, and about 20% of neurons show GABA immunoreactivity (Küppers et al., 2003). The olfactory system has been a site for an extensive study of GABAergic signaling, specifically in the fly AL. Two types of GABAergic neurons (projection neurons and local interneurons) project to the AL (Okada et al., 2009), and application of a GABA receptor agonist inhibits AL function (MacLeod and Laurent, 1996; Stopfer et al., 1997; Sachse and Galizia, 2002).

### GABA Synthesis, Action, and Metabolism

In flies, GABA is synthesized by glutamic acid decarboxylase (GAD) enzymes, including Gad1 (expressed exclusively in the nervous system) and Gad2 (expressed exclusively in glia) (Manev and Dzitoyeva, 2010). GAD is implicated in the formation of synapses at the neuromuscular junction (NMJ) and may also be involved in local regulation of glutamate at NMJ synapses (Featherstone et al., 2000). Researchers have also mapped the expression of Gad1 and Gad2 within the fly brain and found that while only a few neurons release GABA, most of the neurons in the antennal lobe receive inhibitory signals (Okada et al., 2009). GABA exerts action on both ionotropic receptors and GPCRs: ligand-gated GABA_A_-type receptors and metabotropic GABA_B_-type receptors (Hosie et al., 1997). Flies have subtypes of both of these receptors, which are experimentally useful in their sensitivities to different pharmacological manipulations. For example, RDL receptors (GABA_A_-type), named for resistance to the insecticide dieldrin (RDL), are highly distributed in the insect CNS and are therefore the target of numerous insecticides (McGonigle and Lummis, 2009). Importantly, fly GABA receptors do not respond to pharmacological agents the same way that mammalian GABA receptors do, so this will be important to consider when evaluating the relationship between alcohol and the fly GABAergic system. GABA action terminates in the synapse through a variety of mechanisms, such as changes in the density of GABA receptors or GABA uptake by astrocytic GABA transporters (GATs) (Muthukumar et al., 2014). The vesicular GABA transporter (VGAT), located pre-synaptically in GABAergic neurons, packages GABA into synaptic vesicles for later release (Enell et al., 2007).

### GABA and Ethanol in *Drosophila*

In vertebrates and invertebrates, GABA activity impacts numerous behaviors since it is highly expressed in different types of neurons throughout the brain. In *Drosophila*, these behaviors include locomotion (Leal and Neckameyer, 2002), olfactory learning (Liu et al., 2007), and sleep regulation (Agosto et al., 2008). See Table 1 for a summary of GABA roles in fly behavior.

GABA is ubiquitously expressed and involved in regulating numerous behaviors, making it a good target for alcohol, which acts in a widespread, non-selective manner throughout the brain. Alcohol increases GABA release in vertebrates, suggesting a possible role in flies (Kelm et al., 2011). Researchers have particularly focused on the role of metabotropic GABA_B_ receptors in alcohol-related behaviors such as sensitivity, tolerance, and locomotion.

#### Sensitivity and Sedation

Feeding flies the GABA_B_ agonist SKF 97541 increases their sensitivity to sedation when exposed to ethanol vapor (Ranson et al., 2020). These effects persisted for 4 days. Additionally, the SKF 97541-fed flies still developed alcohol tolerance and actually became much more tolerant than controls on the fourth day of exposure (Ranson et al., 2020). When repeating these experiments using the GABA_B_ antagonist CGP 54626, flies became significantly less sensitive to alcohol than controls. However, these manipulations did not affect the development of tolerance, suggesting that GABA_B_ receptors are just one of several receptor systems contributing to tolerance development (Ranson et al., 2020). These results suggest that GABA_B_ receptors mediate ethanol sensitivity and the development of tolerance (Ranson et al., 2020; see Figure 2).

#### Locomotion and Tolerance

Early research on *Drosophila* GABA_B_ receptors involved injecting alcohol into the fly in conjunction with either 3-AMPA, a GABA_B_ agonist, or CGP 54626. Both ethanol and 3-AMPA caused immobility in the fly when injected initially; however, injecting flies with CGP 54626 before ethanol lessened the effects significantly (Dzitoyeva et al., 2003). This data suggests that GABA_B_ activation mediates the behavioral outcomes of 3-AMPA and ethanol. Rapid ethanol tolerance was inhibited by pretreatment with the GABA_B_ agonist, while the antagonist did not impact tolerance (Dzitoyeva et al., 2003). These findings seem to contradict the previously mentioned experiments done by Ranson et al., but may be explained by the length of the study since Ranson et al. did not note significant development of tolerance until the third day of testing and were likely assessing chronic rather than rapid tolerance (Ranson et al., 2020), while Dzitoyeva et al. only noted tolerance for the first 18 h after treatment (2003).

Gamma-hydroxybutyric acid (GHB) also affects ethanol-related behaviors. GHB is a GABA metabolite with medical applications and pharmacological similarities to ethanol. It is also a possible treatment for AUD (Poldrugo and Addolorato, 1999). GABA_B_ receptors mediate the behavioral effects of GHB in flies, providing helpful information for a better understanding of ethanol-related behaviors. Prior exposure to ethanol reduced GHB-associated effects on alcohol sensitivity. However, this tolerance did not occur in the inverse, suggesting that while both GHB and alcohol involve GABA_B_ receptors, their sites or mechanisms of action may differ (Dimitrijevic et al., 2005).

## Acetylcholine

In *Drosophila*, ACh is broadly expressed (Buchner, 1991) and is the primary excitatory neurotransmitter, but despite this, little is known about specific outcomes of ACh signaling for fly behavior. In part, this gap in knowledge arises from the highly detrimental nature of systemic manipulation of the fly cholinergic system. Because ACh is so prevalently expressed, perturbations to ACh signaling result in severe behavioral outcomes (like seizures) that are not favorable for survival (e.g., Somers et al., 2018). The further development of genetic tools targeting more specific cell populations will facilitate an increased understanding of the role of ACh. Kenyon cells, the MB intrinsic neurons, contain ACh-processing proteins. Also, cholinergic activity in these cells impacts activity of MB output neurons (MBONs) (Barnstedt et al., 2016). Kenyon cells exclusively use ACh for intercellular communication, supporting ACh's excitatory role in the fly CNS (Shih et al., 2019).

### Acetylcholine Synthesis, Action, and Metabolism

ACh is derived from choline, which flies ingest through the diet. Choline acetyltransferase (ChAT) catalyzes ACh biosynthesis, and acetylcholinesterase (Ace) breaks down ACh. There are two categories of ACh receptors: ionotropic (nicotinic) and metabotropic (muscarinic). Nicotinic ACh receptors (nAChRs) in *Drosophila* mediate fast, excitatory synaptic currents (Su and O'Dowd, 2003). Muscarinic ACh receptors (mAChRs) are not as well-understood, although researchers have identified three types (A, B, and C) that signal via activation of different G-protein subunits to initiate various downstream intracellular processes (Collin et al., 2013; Ren et al., 2015). Once synthesized presynaptically, ACh must be loaded into vesicles by the vesicular ACh transporter (VAChT) (Kitamoto et al., 1998). While AChE normally terminates ACh action in the synaptic cleft, some drugs prevent this process. Inhibiting AChE is lethal, so irreversible AChE inhibitor compounds are extremely toxic and often used as insecticides (Menozzi et al., 2004). Irreversible AChE inhibitors are also lethal to humans, although reversible AChE inhibitors have some therapeutic applications, like as pharmacological treatments for neurodegenerative diseases, including Alzheimer's (Colović et al., 2013).

### Behavioral Effects of Acetylcholine

Although we know relatively little about cholinergic effects on *Drosophila* behavior, olfactory associative learning is one area of investigation. Silva et al. examined the role of mAChR-A in aversive learning. Researchers generated fly lines to visualize mAChR-A with GFP, and they confirmed mAChR-A expression in the MBs (Silva et al., 2015). By disrupting mAChR-A pharmacologically or genetically, they significantly impaired the formation of aversive olfactory memory in *Drosophila* larvae and adult flies (Silva et al., 2015). However, when flies received a more intense shock during training (90V compared to 50V), learning was not impacted. Thus, mAChR-A may only reinforce moderate aversion (Bielopolski et al., 2019). These effects were localized to mAChR-A activity in the adult gamma Kenyon cells, showing that aversive olfactory learning and short-term memory require mAChRs (Bielopolski et al., 2019).

Investigators also examined olfactory learning in ionotropic AChRs. Mutant flies with disrupted nAChRs in the MBONs showed a reversal in odor driven behavior. They approached an aversive odor, establishing a role for nAChR subunits in the MBONs in olfactory behaviors (Barnstedt et al., 2016). In another experiment on naïve avoidance utilizing the same aversive odor, knockdown of mAChR-A did not impact naïve avoidance (Bielopolski et al., 2019), suggesting that ionotropic receptors specifically mediate this behavior.

Although little research exists regarding ACh's role in alcohol-related behaviors in *Drosophila*, other drugs of abuse have been examined, such as nicotine. Although nicotine's mechanisms of action and behavioral outcomes differ from those of alcohol, both are associated with a period of elevated mood and increased activity at low doses and aversive effects at higher doses (Little, 2000). Additionally, in humans, use of both substances may arise for similar reasons and share similar patterns of use and abuse (Little, 2000). Nicotine has known effects on neurotransmitter systems. When flies are exposed during development, there are fewer TH-positive neurons in the PPM3 cluster of the adult brain, suggesting dopaminergic impacts (Morris et al., 2018). Nicotine exerts direct effects on the cholinergic system by activating nAChRs and producing fly behavioral responses such as hyperactivity (Ren et al., 2012), disrupted geotaxis (King et al., 2011), and loss of startle response (Fuenzalida–Uribe et al., 2013). These behavioral alterations are similar to those we have discussed in reference to alcohol. Also, in probing these effects at various developmental stages for *Drosophila*, research shows that fly responses to nicotine are comparable to mammals throughout development (Velazquez-Ulloa, 2017). Therefore, nicotine research in *Drosophila* may provide a useful point of reference for future explorations of alcohol and ACh.

Because it is the primary excitatory neurotransmitter, ACh activation drives the downstream release of neuromodulators like DA and OA. nAChR activation with pharmacological agents led to a rapid, dose-dependent release of OA (Fuenzalida–Uribe et al., 2013). In a startle-induced negative geotaxis assay, flies exposed to nicotine do not rapidly climb up the vial following a mechanical disruption that taps them to the bottom of the tube. However, disrupting OA transmission abolished the nicotine-induced impairment of flies' startle response, returning negative geotaxis to normal. Therefore, the behavioral response to drugs like nicotine involves AChR-induced OA release (Fuenzalida–Uribe et al., 2013).

## Glutamate

The glutamatergic system in the *Drosophila* brain presents a bit of a mystery. While glutamate is one of the best characterized neurotransmitters in the mammalian brain, it is one of the least understood for the fly. In the mammalian CNS, glutamate is the primary excitatory neurotransmitter, but this is not true for flies. Studies show that there are numerous glutamatergic neurons distributed throughout the adult fly CNS (Daniels et al., 2008; Raghu and Borst, 2011), and glutamate is well-established as the primary excitatory neurotransmitter at the NMJ (Jan and Jan, 1976). However, its role in brain activity remains somewhat enigmatic. Because glutamate exerts excitatory effects at the NMJ and in the vertebrate CNS, investigators have considered its excitatory potential in *Drosophila*. Studies of ionotropic glutamate receptor (iGluR) subunits with homology to vertebrate receptors have not established conclusive excitatory mechanisms in the fly brain, and, in fact, glutamate may be inhibitory in some circuits, like for olfaction (Liu and Wilson, 2013). Specifically, in the olfactory system, glutamatergic inhibition is mediated by the glutamate-gated chloride channel (Liu and Wilson, 2013), and the gene encoding this channel, GluClα, also mediates glutamatergic inhibition in the fly visual system (Molina-Obando et al., 2019).

### Glutamate Synthesis, Action, and Metabolism

Glutamate is an amino acid that is produced by neuron-glia interactions in the glutamate-glutamine cycle, which involves the enzymes glutamate dehydrogenase (Gdh) and glutamine synthetase (GS) (Vernizzi et al., 2019). Gdh converts glutamate to alpha-ketoglutarate and ammonia (Plaitakis et al., 2017), and GS is a cytosolic enzyme that produces glutamine (Spodenkiewicz et al., 2016). Cytosolic glutamate is a precursor in the synthesis of GABA (Daniels et al., 2008). The fly genome contains 30 iGluR subunits (Littleton and Ganetzky, 2000), one metabotropic receptor (Mitri et al., 2004), and one glutamate-gated chloride channel (Cully et al., 1996), suggesting that glutamate can exert numerous effects in the fly brain. The vesicular glutamate transporter (VGlut) fills synaptic vesicles with glutamate. There is a single *Drosophila* VGlut found on synaptic vesicles at the NMJ at synapses on motoneurons and interneurons throughout the CNS (Daniels et al., 2004).

### Behavioral Effects of Glutamate

Because glutamate in isolation has historically been challenging to study outside the NMJ, it is not well-understood what behavioral effects glutamate is uniquely involved in regulating. Glutamatergic activity is better characterized in vertebrates, and these findings provide foundations for studying the role of glutamate in behavior for *Drosophila* as well. In vertebrates, much of our understanding of glutamatergic activity comes from understanding the action of the three iGluRs: AMPA, kainate, and NMDA receptors (Traynelis et al., 2010). Although *Drosophila* iGluRs share sequence similarity with the vertebrate receptors, subsequent Investigations have been somewhat limited because invertebrate neurons are small and challenging to access, complicating further characterization of these receptors' functions (Li et al., 2016a).

In mammals, glutamatergic activation likely has circadian fluctuations (Prosser, 2001), which is also true for flies (Zimmerman et al., 2017). Reducing glutamatergic release from glutamatergic neurons decreased wakefulness, and increased glutamatergic activity promoted wakefulness (Zimmerman et al., 2017). These results suggest that glutamate is wake-active in *Drosophila* (Zimmerman et al., 2017). Optogenetic studies indicate that the dorsal population of circadian clock neurons use glutamate as an inhibitory transmitter to promote sleep. This signaling may play a particularly significant role in daytime sleep (Guo et al., 2016). See Table 1 for a summary of glutamate roles in fly behavior.

Glutamate likely plays an important role in the antennal lobe. The AL is known to contain both ionotropic and metabotropic glutamate receptors, including the fly homolog of the NMDA receptor, Nmdar, which is thought to have a conserved function in synaptic plasticity and, therefore, olfactory learning and memory (Xia et al., 2005). In the fly olfactory circuit, glutamate is a neurotransmitter with inhibitory effects, primarily influencing the response that projection neurons in the AL have to olfactory stimuli (Liu and Wilson, 2013). Interrupting glutamatergic transmission by expressing an RNAi transgene for one of the NMDA receptor subunits, *Nmdar1*, reduces Nmdar1 receptor subunit levels. Reducing Nmdar1 activity in a subset of AL projection neurons responsive to a specific odor blocked short and long-term olfactory habituation to that specified odor without impacting habituation to other odors (Das et al., 2011). Additionally, RNAi knockdown of Gad1 or VGlut in AL local interneurons blocks short and long-term olfactory habituation, suggesting that both GABAergic and glutamatergic activity in local interneurons are important for habituation (Das et al., 2011).

There is little research focused specifically on glutamate's role in ethanol-related behaviors in *Drosophila*. In olfactory receptor neurons of the AL, a single alcohol exposure induces excitotoxic cell death via glycogen synthase kinase-3 beta and NMDA receptors, suggesting a glutamatergic role for alcohol-induced neural deficits in the fly (French and Heberlein, 2009). Some recent evidence suggests that a circuit involving DAergic modulation of glutamatergic MBONs is involved in consolidation and expression for alcohol-associated memories (Scaplen et al., 2020). Two DAergic projections are to glutamatergic MBONs implicated in arousal (Sitaraman et al., 2015; Scaplen et al., 2020). DAergic activity may inhibit these MBONs, permitting consolidation of alcohol preference. These effects provide insight into neural mechanisms for the association of alcohol with context cues and memories, which is a critical feature of the persistence of addiction behaviors (Scaplen et al., 2020). As genetic and behavioral tools become increasingly advanced, this area of research should continue to expand.

Glutamate is unique in that it has a multi-faceted role in the fly brain, exerting both excitatory and inhibitory effects (Jan and Jan, 1976; Liu and Wilson, 2013). Furthermore, the complex action of poorly understood neurotransmitters like glutamate and ACh is complicated by phenomena such as dual neurotransmission. Dual neurotransmission overrides the classical view of “one neuron, one transmitter” and shows that neurons often release two or more neurotransmitters (Vaaga et al., 2014). The majority of OA neurons in the *Drosophila* brain are also glutamatergic, and dual neurotransmission is involved in behaviors like aggression and courtship (Sherer et al., 2020). These discoveries have been made possible via new tools enabling detailed glutamatergic manipulations like RNAi knockout of glutamate in OA neurons (Sherer et al., 2020) and circuit tracing and labeling with trans-Tango (Talay et al., 2017). Other developments include the electrophysiological characterization of neurons (Liu and Wilson, 2013), GAL4/UAS inhibition of specific glutamatergic neurons (Liu and Wilson, 2013), and post-synaptic knockdown of glutamate receptors (Das et al., 2011). Using these glutamatergic advancements as a model, outcomes for other complex and overlapping neurotransmitter systems will continue to be clarified.

## Conclusion

Twenty years ago, little was known about the role of DA in fly behavior, and it was an area receiving relatively little research focus. However, we have now identified that even though there are only about 250 DA neurons, many small subsets of these impact distinct behaviors (e.g., Kong et al., 2010), and DA is an area of high interest to researchers studying *Drosophila* behavior. These discoveries have been made possible through tools like split-GAL4 lines that enable overexpression or knockdown of genes in specific subsets of neurons, and CRISPR/Cas9 mutagenesis, which allows for the targeted investigation of a mutated gene. Tissue-specific CRISPR has also been applied in *Drosophila* to restrict mutagenesis to a particular subset of cells (Meltzer et al., 2019; Poe et al., 2019; Port et al., 2020). Additionally, researchers recently began to define the *Drosophila* chemoconnectome, which comprises all neurotransmitters, neuromodulators, neuropeptides, and their receptors (Deng et al., 2019). The chemoconnectome has been made possible by advancements in genetic manipulation and neural mapping. As it is expanded upon, it will provide an invaluable resource for describing neurotransmitters anatomically and functionally. As tools continue to increase in precision and sensitivity, we will further unravel the roles of little-explored neurotransmitters in alcohol-induced behaviors. The future is bright for this area of research, and discoveries are undoubtedly imminent.

*Drosophila* is genetically tractable and displays a huge behavioral repertoire, making it an extremely useful model organism for neuroscience. Recent years have brought advancements in genetic and behavioral tools that make flies increasingly advantageous. *Drosophila* are an especially suitable candidate for studying behaviors like alcohol response, which is challenging to investigate in mammals due to alcohol's widespread action throughout the brain. No organism has a specific, unique circuit or receptor for alcohol, so it must be explored in reference to its impacts on the various existing biological pathways of which it takes advantage. Alcohol-induced neurotransmitter modifications and associated influence on behavior are one critical tool for unraveling the neurobiological effects of alcohol. In manipulating fly neurotransmitter systems and assessing impacts on ethanol-related behaviors, we further make sense of the complicated relationship between brain and behavior relating to alcohol.

## Author Contributions

MC wrote the manuscript with input from IT and AR. All authors contributed to the article and approved the submitted version.

## Conflict of Interest

The authors declare that the research was conducted in the absence of any commercial or financial relationships that could be construed as a potential conflict of interest.
